# Qualitative evaluation of a local coronary heart disease treatment pathway: practical implications and theoretical framework

**DOI:** 10.1186/1471-2296-13-36

**Published:** 2012-05-14

**Authors:** Lena Kramer, Kathrin Schlößler, Susanne Träger, Norbert Donner-Banzhoff

**Affiliations:** 1 Department of General Practice/Family Medicine, University of Marburg, Karl-von-Frisch-Straße 4, 35043, Marburg, Germany

**Keywords:** Primary health care, Coronary artery disease, Clinical pathways, Qualitative research

## Abstract

**Background:**

Coronary heart disease (CHD) is a common medical problem in general practice. Due to its chronic character, shared care of the patient between general practitioner (GP) and cardiologist (C) is required. In order to improve the cooperation between both medical specialists for patients with CHD, a local treatment pathway was developed. The objective of this study was first to evaluate GPs’ opinions regarding the pathway and its practical implications, and secondly to suggest a theoretical framework of the findings by feeding the identified key factors influencing the pathway implementation into a multi-dimensional model.

**Methods:**

The evaluation of the pathway was conducted in a qualitative design on a sample of 12 pathway developers (8 GPs and 4 cardiologists) and 4 pathway users (GPs). Face-to face interviews, which were aligned with previously conducted studies of the department and assumptions of the theory of planned behaviour (TPB), were performed following a semi-structured interview guideline. These were audio-taped, transcribed verbatim, coded, and analyzed according to the standards of qualitative content analysis.

**Results:**

We identified 10 frequently mentioned key factors having an impact on the implementation success of the CHD treatment pathway. We thereby differentiated between pathway related (pathway content, effort, individual flexibility, ownership), behaviour related (previous behaviour, support), interaction related (patient, shared care/colleagues), and system related factors (context, health care system). The overall evaluation of the CHD pathway was positive, but did not automatically lead to a change of clinical behaviour as some GPs felt to have already acted as the pathway recommends.

**Conclusions:**

By providing an account of our experience creating and implementing an intersectoral care pathway for CHD, this study contributes to our knowledge of factors that may influence physicians’ decisions regarding the use of a local treatment pathway. An improved adaptation of the pathway in daily practice might be best achieved by a combined implementation strategy addressing internal and external factors. A simple, direct adaptation regards the design of the pathway material (e.g. layout, PC version), or the embedding of the pathway in another programme, like a Disease Management Programme (DMP). In addition to these practical implications, we propose a theoretical framework to understand the key factors’ influence on the pathway implementation, with the identified factors along the microlevel (pathway related factors), the mesolevel (interaction related factors), and system- related factors along the macrolevel.

## Introduction

Despite the declining mortality of patients in recent years with coronary heart disease (CHD) in Western countries [1,2], CHD remains the leading cause of morbidity and mortality in adults worldwide [3]. In the United States, about 7.0 % (women) to 9.1 % (men) of the general population are affected by CHD [4]. Similar prevalence rates (women: 6.5 %, men: 9.2 %) have been found in Germany [5]. Most patients with CHD need lifelong, continuous, complex medical care, which is extremely costly to the healthcare system [6].

In this context, an important role is attached to the shared care of patients with CHD by the general practitioner (GP) and the cardiologist. Better coordination and communication between GP and medical specialist promises optimized medical treatment along with increased cost-effectiveness in primary care [7-9]. Improving the cooperation between different health care professionals and providing optimal evidence-based medical care for patients are the main objectives of clinical guidelines [10]. For CHD, it was demonstrated that non-adherence to medical recommendations is associated with a broad range of adverse outcomes in patients [11]. Despite the high scientific quality of most guidelines and their wide promulgation, their actual impact on clinical practice and quality of care is limited [12-15]. Numerous international studies have shown that effective and lasting behaviour change of health care professionals is difficult to achieve and is influenced by multiple factors [16-18]. Although guidelines may be seen as necessary to provide valid recommendations, they are insufficient in ensuring evidence-based decision-making [19]. In this context, the use of clinical pathways as one approach to facilitate the adaption of research findings in daily practice is important. Clinical pathways are multidisciplinary, locally translatable, and involve a stepwise procedure, determined timeframes, and standardized care for a specific clinical problem [20]. Even though the implementation of clinical pathways faces similar problems as the implementation of guidelines [21,22], some authors [19,23,24] expect treatment pathways to raise implementation chances by adapting the guideline recommendations to local conditions and thereby referring more to physicians’ work reality. However, the effect of local treatment pathways is controversial, as Salegy and colleagues [25] rarely found an implementation benefit by the local adaption. Additionally, the local adaption was associated with higher costs compared to national guidelines. Nevertheless, the development and use of a local pathway might be appropriate in situations where an additional advantage is expected by its use. This might be of importance if system related factors require local adjustments [25].

To date, most experience with treatment pathways is gained in countries with a Beveridge type of health care system, characterized by a strong governmental influence. In countries like Germany, where a Bismarck type of health care system is established [26], the implementation of shared care pathways poses a special challenge as they are less regulated by institutional standards. Patients have universal medical access with only a very limited gate-keeping role of the GP. Due to the system structure, competition between medical professionals in the ambulatory sector (primary and secondary care) hinders cooperation. Thus, despite a greater need for coordination, the establishment of shared care pathways is paradoxically much more difficult to achieve in Bismarck types of health care systems. By developing and implementing a shared care pathway for patients with CHD in a Bismarck type system, we intended to close this gap and contribute a new aspect to existing research primarily made in the Beveridge type of health care systems.

Within this context of developing and evaluating a local CHD pathway we pursued two objectives in this study. On the one hand we aimed to evaluate GPs’ opinion regarding the pathway and give practical implications for clinical practice that derived from this evaluation. On the other hand we strived to abstract our findings by putting the identified key factors influencing the pathway implementation into a multi-dimensional model.

## Methods

### Development and description of the CHD pathway

In 2008, GPs and cardiologists from the Marburg region, Germany, were invited by the Department of General Practice at the University of Marburg to jointly develop a local treatment pathway for patients with CHD. By involving end-users in the pathway development and thus considering their experience and expertise of daily practice, we realized a bottom-up approach [27]. It was assumed that a cooperatively developed local consensus with the collaboration of GPs and cardiologists would improve GPs’ and cardiologists’ acceptance and adherence to the corresponding guideline recommendations for the handling of CHD patients in primary care [19].

The development of the pathway was based on current regional [28] and national care guidelines [29]. Within small working groups moderated by members of the department, plans for monitoring visits, relevant drugs, and documentation forms were developed and brought to a consensus with all participants. To support the implementation of the pathway in daily practice, we provided the physicians with both a laminated pocket version of the pathway guidelines covering drugs and monitoring visits, and patient treatment logs to list medication and monitoring visits for every patient. An overview of the pocket version of the pathway is given in Additional file 1.

### Study design

**Figure 1:**
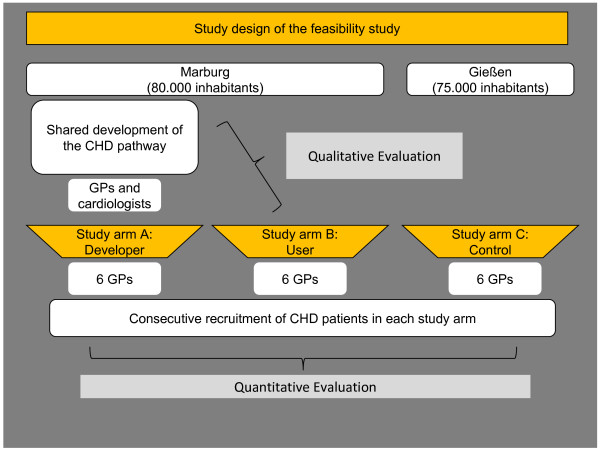
Study design of the larger feasibility study.

Our qualitative study was part of a larger feasibility study [30] with 18 GPs in three study arms (8 pathway developers, 6 pathway users, 6 control group) and 290 consecutively recruited patients with CHD. This larger study was on the development and evaluation of a local CHD treatment pathway using quantitative and qualitative components (mixed methods study). An overview of the intended larger feasibility study design is given in Figure 1.

In brief, the larger project aimed to improve the shared care of patients with CHD by GPs and cardiologists. The quantitative study investigated physicians’ adherence to pathway recommendations regarding the prescription of drugs and the referrals to the cardiologist, and its impact on patient variables such as satisfaction with the treatment and health-related quality of life. The analysis of the quantitative results and its relation to the qualitative data (triangulation) is in progress and will be reported soon. To gain further insight into GPs’ opinion regarding the pathway and the factors influencing GPs’ decision to (not) implement the CHD pathway, we conducted this qualitative study in the middle of the feasibility study so that physicians’ experience with the pathway could be ascertained. Face-to-face interviews were undertaken during February and March 2010 in the physicians’ practices by one of the authors (LK) who was not involved in the development of the pathway.

In our study we aimed not only to report the results of the interviews and their practical implications, but to identify the latent pattern of the data. According to Sandelowski and Barroso, this more explanatory than exploratory interpretation of the qualitative data can be categorized as thematic/conceptual as we provide a theoretical framework of our findings [31]. The results gained in our study, like those of the quantitative study, may inform the planning and conducting of an subsequent randomised controlled trial (RCT) evaluating the efficacy of the CHD pathway [30].

GPs and patients were informed in detail about the study and all gave their written consent to the study participation. Ethical approval for the study was obtained from the Ethics Committee of the Faculty of Medicine at the Phillips University of Marburg, Germany.

### Participants and recruitment procedures

Within the context of the feasibility study, we asked all 12 pathway developers (8 GPs and 4 cardiologists) and the 6 pathway users, familiar with the pathway recommendations, to participate in our qualitative evaluation study. Thus, by selecting respondents that were most likely to yield useful information about the key factors influencing the pathway implementation, we used a purposeful sampling strategy [32] and considered our sample size as appropriate to achieve saturation [33]. As the GPs of the control group did not know the pathway, we did not include them in the study. All physicians from the intervention groups were located in the Marburg region, Germany, and were recruited from the regional physician network of the Department of General Practice at the Phillips University of Marburg, Germany. The GPs from the control group were located in a neighbouring town.

### Data collection

The interviews were based on a semi-structured interview guideline covering the attitude towards the developed pathway and medical innovations such as treatment pathways and guidelines in general, key factors that influenced the implementation of the CHD pathway in a negative or positive way, and suggestions of improvement and behaviour changes in consequence of the pathway. The interview guideline was developed jointly by two of the authors (LK, NDB) and was aligned with previously conducted studies from our department on changes of professional behaviour [34,35] and the components of the theory of planned behaviour (TPB) [36]. Interview participants were assured that their responses would remain confidential and anonymous.

### Data analysis

Interviews were audio-taped and transcribed verbatim. The accuracy of transcripts was checked prior to being transferred to the computer software program Maxqda 2007 [37], which assisted data handling. Based on the interview guideline, a thematic coding frame for the analysis of the interviews’ content was developed by the authors (LK and KS). The first three interviews were coded and then the evaluation of the coding frame was discussed. Within the analysis process no further categories emerged, so we considered theme saturation as achieved. Transcripts were coded separately by LK and KS and then checked for consistency. The data analysis and interpretation followed the standards of qualitative content analysis [38]. The translation of the quotes from German to English was conducted by a qualified translator.

## Results

### Study population

Twelve pathway developers (8 GPs, 4 cardiologists) and four pathway users consented to conducting face-to-face interviews. The reason for non-participation of 2 GPs in the user group was a lack of time. The majority of the interviewees were male (75 %), between 41 and 50 years of age (56 %), and employed in full-time practice (94 %). Most of the interviewed GPs and cardiologists (75 %) strongly agreed with the pathway. Further characteristics of the participants are summarized in Table 1.

**Table 1:** Demographics of the study sample: GPs and cardiologists (n = 16)

**Demographics and professional characteristics**	**n**	**(%)**^**a**^
**Gender**		
Male	12	(75.0)
Female	4	(25.0)
**Age (years)**		
≤ 40	1	(6.3)
41 to 50	9	(56.3)
51 to 60	5	(31.3)
> 60	1	(6.3)
**Established since (years)**		
≤ 10	10	(62.5)
11 to 20	1	(6.3)
> 21	5	(31.3)
**Characteristic of the practice**		
Single practice	5	(31.3)
Group practice	11	(68.8)
**Practice location**		
< 5000	5	(31.3)
5000 to 20.000	2	(12.5)
20.000 to 100.000	9	(56.3)
**Status**		
Full time	15	(93.8)
Part time	1	(6.3)
**Agreement with the treatment pathway**		
Strong agreement	14	(87.5)
Average agreement	2	(12.5)

^a^ Percentages may not add up to 100 % due to rounding.

### Key factors influencing the (non-)implementation of the treatment pathway

As shown in Table 2, we summarized our codings systematically in subordinated key factors and assigned them to a pathway-related (pathway content, effort, individual flexibility, ownership), behaviour-related (previous behaviour, support), interaction-related (patients, shared care/colleagues) or system-related main category (context, health care system). In total, we identified 10 key factors influencing the implementation of the pathway.

**Table 2:** Classification of the key factors influencing the pathway implementation

**Codings**	**Key Factors**	**Main Category**
· Content	Pathway	Pathway
· Layout	material	
· Assessment		
· Communication	Ownership	
· Assessment		
· Time	Effort	
· Organisation		
· Pathway availability		
· Assessment of guidelines	Individual	
· Application area	flexibility	
· “Cookbook medicine” vs. individuality		
· Improvements of implementation strategies		
· Motivation		
· Behaviour change	Previous	Behaviour
· Sustainability	behaviour	
· PC Version	Support/	
· Training	Reminders	
· Background	Patient	Interaction
· Relationship		
· Compliance		
· Communication	Colleagues/	
· Cooperation	Shared Care	
· Geography	Context	System
· Cooperation		
· Disease Management Program (DMP)		
· Bureaucracy	Health care	
· Health care system	system	

As this study was undertaken to gain additional insight into the relevant key determinants for implementation, we focused a cross-case analysis on the identification of general factors and were less interested in interindividual differences.

### Pathway related factors

#### Pathway content

The first major theme in this category dealt with the pathway content. Many of the participating GPs and cardiologists (C) considered the CHD pathway as a useful and high quality treatment aid.

“The treatment pathway is good to create a standard framework of how to interact with certain patients, so that it is clear for everybody. I think it’s a reasonable thing.” [C1]

“I think it’s actually useful to have such an aid. It improves your awareness for things you should keep an eye on. To my delight, the number of beta blockers was reduced and more certainty was provided regarding the sequence of cardiological consultations.” [GP2]

#### Effort

The effort created by the implementation of the pathway in daily practice was perceived as controversial by the interviewees. While the case record form (CRF) documentation for the evaluative study was assessed as time consuming, the application of the pathway in practice was rated as time saving, or no difference was recognized.

“It was time consuming to somehow record [the patients’ medication history]. The second time around it was no problem because in most cases nothing had changed. You could copy [the information] or just refer to the first record. Integrating [the information] is not a problem now, unlike the first time.” [GP6]

“I would say that [the pathway] certainly saves time, [especially] for colleagues who haven’t followed [the pathway’s recommendations] or have yet to really put the drug therapy into practice.” [GP1]

#### Individual flexibility

A relevant issue for the participating physicians was the balance between ‘cookbook medicine’ by a high adherence to the pathway’s recommendations and an individual treatment of the patient. On the one hand, participants appreciated the standardised treatment guideline, on the other hand, they emphasized the consideration of intuition and experience in the consultation.

“It is indeed like cookbook medicine, but on the other hand, it makes one feel safer. And I think that every one of us has a certain plan of how to proceed for every clinical situation. And those [plans] could be standardized. Then you can say ‘Ok, we consistently proceed according to this model, to this scheme.’ I think this is good.” [GP7]

“As a doctor, I want to maintain individual treatment. I am the one who decides based on my twenty years of experience in cardiology. I do not want the pathway to tell me what I have to do. Guidelines are only guide rails within which you can act.” [C1]

#### Ownership

The participation in the development of the pathway was an important factor for the physician’s evaluation of the CHD treatment tool. Overall, the participation resulted in a positive evaluation, even though concessions had to be made.

“Our behaviour informed the pathway, like always. I support [the pathway]; we worked on [its development] for a long time. It was extensively discussed before everybody could agree on its final version. So I perceived [the pathway development] as positive.” [C2]

“You are not bound to a guideline that was imposed from above, but in whose development we took part, and which we could influence. This is what makes it good. If you contribute to such a thing, then it is something you support. My ideas are included and the way I think you should act. And, therefore, I am totally in support of it.” [GP1]

“We could not enforce [the frequency of cholesterol measurement]. When you make decisions as a group you have to swallow some bitter pills.” [C3]

### Behaviour-related factors

#### Previous behaviour

The appraisal of their own behaviour in the past was an important factor for physicians’ willingness to implement the pathway. Many interviewees felt that they had treated their patients with CHD according to the pathway recommendations during previous visits.

“[Our behaviour] has not changed a lot. We have not invented something new, but have, in principle, implemented the guidelines.” [GP5]

“Actually, I acted like I always did: the patients came in regular intervals, we talked about the disease, we talked about incidents, we talked about the cardiological report, and we checked the laboratory parameters and drugs.” [GP6]

#### Support/Reminders

The participating GPs expressed a desire to integrate the pathway recommendations into practice software. A PC integration was perceived as facilitating the embedding of the pathway in daily practice, e.g., the schedule for monitoring visits. Concerning the need for regular refresher meetings, the physicians had heterogeneous opinions: On the one hand the knowledge update was appreciated; on the other hand regular meetings were seen as too time-consuming or simply unnecessary.

“Of course we would keep recommendations in mind with regular training sessions, but we already have so many continuing medical education (CME) events. So it is not necessary.” [GP3]

“I think it’s reasonable to have regular trainings so that it becomes second nature [to us doctors]. I think this is very important.” [GP1]

### Interaction-related factors

#### Patient

Patients’ acceptance of and compliance with the pathway recommendations were rated high by the interviewees if the pathway recommendations were thoroughly explained. Furthermore, the participating physicians reported that knowledge of the patient’s background, e.g., medical history and psychosocial conditions, may influence the application of the pathway. This can be reflected in either patient related assumptions or routine actions in the consultation.

“And for the patient it [the pathway] is actually better. He stays more compliant because he knows it is not back and forth but a routine program that he gets every time. This makes the patients feel safer.” [GP8]

“Of course we act according to the disorders and the risk profile, but we know the patients from their domestic conditions and whether they comply or not. And in these cases I would vary [the treatment].” [GP9]

“Being aware gets lost sometimes, especially when you have had patients for years or take over a practice and there are listed diagnoses and drugs, so you do not always question everything, you just overlook things.” [GP2]

#### Shared care/Colleagues

The cooperation between GPs and cardiologists was appreciated by both sides. Apart from smaller disagreements concerning coordination responsibility for the CHD patients, or adherence to the pathway advice, the collaboration was described as positive.

“We actually understood it as the treatment pathway helps us to do our job, and that one part of that - what we have done before - is connected to the primary care treatment. If we collaborate with competent GPs, we already tend to tell our patients that they have a competent GP to whom they should primarily turn to, e.g., when deterioration happens.” [C3]

“No problem now, [the cooperation] has improved considerably. The cardiologists do not make it their responsibility to schedule monitoring visits, instead leaving it more to the GP`s discretion. [Cardiologists] complained that many [patients] come to unnecessary monitoring visits, but now I think it is working better.” [GP10]

### System-related factors

#### Context

By integrating the pathway into a larger geographic, such as nationwide or organizational contexts, e.g., a medical association, the interviewed physicians expected an increased implementation success of the tool. The overlap between the pathway and the German CHD Disease Management Program (DMP) as systematic treatment program for chronically ill people [6,39] was seen as an impeding factor for the pathway implementation in cases of differing recommendations.

“But I think that one can and should implement [the pathway] at the health care system level. One could, for example do [the pathway implementation] with our medical association and raise it to another level.” [C2]

“Because we are bound by contract to the DMP, we are obligated to act correspondingly. Even if we found the treatment pathway more reasonable, we are forced to implement the DMP. Sanctions are partially imposed if you do not participate in the DMP.” [GP7]

#### Health care system

Participants perceived the actual demands of the German health care system as influencing the implementation of the CHD pathway. Due to competing tasks in daily practice, the pathway would be at risk of being forgotten. The GPs named their small gate-keeping influence in the German health care system as influencing factors for appointments with medical specialists as patients have free access to specialised care.

“We have so many fires burning at the moment, so many contracts, special regulations and treatment plans, DMP situations and such, that we partly lose the big picture. That makes it difficult for us.” [GP7]

“Thus, there is so much to work on at the moment. I think that is the main reason for not optimally meeting the requirements of every patient.” [GP9]

“I actually see another essential problem in, such is it here, we have free choice of care and many people go to secondary care on their own decision.” [GP7]

## Discussion

### Main findings

Our study complements the understanding of potential facilitators and barriers to be considered in planning the implementation of innovative tools like the CHD pathway and is in line with the findings of other international studies [10,16,17,40].

Concerning the treatment pathway itself, we first assessed the pathway content, the participation in the pathway development/ownership, the individual flexibility, and the related effort as relevant aspects for the implementation. The overall evaluation of the pathway recommendations was positive and required no additional effort by some of the GPs. Referring to the pathway development, which was appreciated by the physicians, a study by Grimshaw and colleagues showed that the participation in developing a guideline could enhance the compliance in physicians by about 32 %, whereas it was 22 % for non-developers [12]. Additionally, the active involvement of end-users in the local adaptation of clinical guidelines was found to lead to lead to significant changes in practice through a sense of ownership [19,41]. As seen in other studies, the interviewed physicians perceived the ambivalence between the facilitation by standardized guidance and the restriction of their own medical autonomy as relevant [42-44].

In addition to the aspects related to the pathway, we identified further relevant factors. On a behaviour-related level we identified previous behaviour and support as important factors for pathway implementation. Located on an interactional level and consistent to other research, the patients’ acceptance of the pathway and the physicians’ cooperation with their colleagues were assessed as important components for the implementation of the pathway [45]. Patients’ acceptance of the recommendations of the pathway was rated high by the physicians. In accordance with a study by Summerskill and Pope, our findings suggest that a physician’s knowledge of a patient’s background is an influencing factor for the successful implementation of medical innovations [45]. As a result, this could lead to assumptions, which then exclude or include specific treatments for certain patients, for example those with a certain risk profile. Contrarily, physicians’ knowledge of a patient’s background may also result in routine actions in the consultation, which may lead to overlooking other indications.

Finally, on a system level, an effective use of the CHD pathway depends on both the health care system and the context in which the medical implementation occurs.

Considering the impact of contextual factors for the successful implementation of medical innovations is an aspect also mentioned by other authors [40,46]. We aimed to raise the pathway’s implementation success by realizing a bottom-up approach and local attuning in the development and implementation of the CHD pathway; this can be seen in other studies where the application of a bottom-up strategy led to improvements in outcomes of care [47,48]. In contrast to these findings, our results suggest that physicians also attach importance to enabling features that can be centrally provided, such as embedding the pathway in a DMP, or providing regular trainings.

### Practical implications

Even though the overall evaluation of the pathway recommendations was positive, this appreciation did not automatically lead to a behaviour change as some physicians reported to have already been treating their patients as the pathway recommends. According to the theory of planned behaviour (TPB) [36], attitude is an important factor for behaviour change, but our study reveals that on its own it is an insufficient precondition for a lasting implementation of the pathway. Thus, to enhance the adaptation of the pathway in daily practice, an appropriate implementation strategy is required. As the effectiveness of single measures depends on the context, the professional group or the disease [49], an individually designed strategy that includes different components is thought to be most promising [50]. A first step to raising the awareness of the physicians might be to modify the layout of the laminated pocket version as a pathway-related factor. Another approach facing interactional and behavioural factors is to establish regular quality circles where the appropriate use of the pathway is discussed and supervised. Some of the interviewed physicians felt that their individual treatment flexibility was limited by the pathway as it proposes a structured treatment plan. Thus, to prevent a complete rejection of the pathway, it might make sense to broaden coarsen the recommendations and give benchmarks instead of detailed instructions. Being aware that GPs’ perception of conformity does not necessarily correspond to real facts, further research must be undertaken to investigate a supposed perception-reality gap.

As mentioned by the physicians, the implementation of the pathway would be eased by its integration into PC software and thus simplify and standardize recall possibilities. Another factor influencing the pathway implementation are competing recommendations of the pathway and the German DMP, as the DMP includes mostly similar, but sometimes aberrant recommendations and different timetables. A possible practical implication would be to embed the pathway recommendations directly in a DMP, or taking the DMP recommendations into account during the process of pathway development.

### Theoretical framework

**Figure 2:**
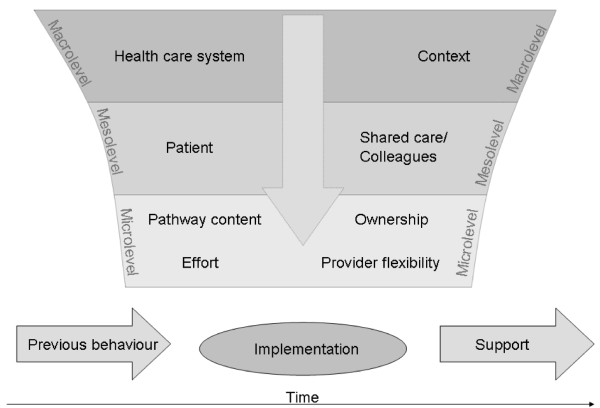
Multi-dimensional model of implementation.

In addition to reporting our findings and giving some practical implications, we aimed to abstract our results and give a theoretical framework (see Figure 2) based on the main categories mentioned in Table 1.

We thereby considered two dimensions: temporal sequence and content. We assumed that the key factors influencing the implementation of the CHD treatment pathway can be classified according to their impact in the pre-intervention, e.g., previous behaviour, the present-intervention, e.g., pathway material, or the post-intervention period, e.g., support. Referring to the Ecological System Theory by Bronfenbrenner [51] and based on a similar model developed in our department [35], we differentiated between several levels of the health care sector, namely the micro-, meso- and macrolevel. Additionally, our model shows similarities with the conceptual framework proposed by Sorensen and colleagues, which addresses the social context (including individual, interpersonal, organizational and neighbourhood/community factors) as essential for behaviour change [52]. Thus, the pathway-related factors were arranged along the microlevel, the interaction-related factors along the mesolevel, and the system-related factors along the macrolevel. Due to their primary time-related impact, the behaviour-related factors were excluded from this content-related classification within the model.

We do not claim completeness of the aforementioned factors or categories, nor do we provide an algorithm of how the presence or absence of the reported key factors attributes to the relative implementation success of the pathway. Our interest was to propose a theoretical framework that might serve as background for further implementation research.

### Study limitations

As a result of the qualitative study design and the small study sample, the representativeness of the data may be limited. In addition, the selection bias caused by the fact that only motivated and interested physicians participated in the study may influence the representativeness of the results. A possible bias by social desirability in the face-to-face interviews cannot be excluded. Nevertheless, due to the meaningful results and conclusions gained in a Bismarck type of health care system, we presume the chosen method to be appropriate for our research question [53-55], which is aimed more at involved components than defined outcome measures [56].

Another limitation concerns the proposed multi-dimensional model. The distinction between the time- and level-related categories should not be seen as static. The model is a draft with flexible confines between the singular categories and has to be proven in further research. For example, due to its actual impact, we related the pathway development to a present point in time; putting this factor in the past would have been another possibility. We did not investigate possible differences in the response patterns of pathway developers and pure users, or GPs and cardiologists, while the research question focused on the identification of general factors influencing the pathway implementation rather than on interindividual differences.

As our research dealt primarily with external implementation factors and final outcomes, the role of underlying psychological and interactional processes will need to be investigated in future studies. In addition to cognitive or affective processes like self-efficacy expectancy [57], general implementation processes and implementation fidelity [58-60] should also be evaluated.

## Conclusions

Our study demonstrated that the implementation of medical innovations like the reported CHD treatment pathway is influenced by multiple components. Besides reporting the factors influencing the pathway implementation and giving practical implications how to improve pathway implementation, we proposed a theoretical framework by arranging the findings in a multidimensional model and thereby add innovative aspects to the already existing implementation research. Fields scarcely investigated collectively in Bismarck type of health care systems, like bottom-up developed local pathways and the integration of primary and secondary care, are considered.

## Competing interests

The authors declare that they have no competing interests.

## Authors' contributions

All authors contributed to study design. LK and NDB developed the interview guideline. LK conducted the interviews. Data coding and analysis were coordinated and performed by LK and KS. LK drafted the manuscript. KS, ST and NDB provided critical review on all parts of the manuscript. All authors read and approved the final version of the manuscript.

## Pre-publication history

The pre-publication history for this paper can be accessed here:

http://www.biomedcentral.com/1471-2296/13/36/prepub

## Supplementary Material

Additional file 1 Pocket version of the CHD treatment pathway. Click here for file

## References

[B1] Levi F Lucchini F Negri E La Vecchia C Trends in mortality from cardiovascular and cerebrovascular diseases in Europe and other areas of the world Heart 2002 88 119 124 10.1136/heart.88.2.119 12117828 PMC1767229

[B2] Kesteloot H Sans S Kromhout D Dynamics of cardiovascular and all-cause mortality in Western and Eastern Europe between 1970 and 2000 Eur Heart J 2006 27 107 113 16204263 10.1093/eurheartj/ehi511

[B3] Lopez AD Mathers CD Ezzati M Jamison DT Murray CJ Global and regional burden of disease and risk factors, 2001: systematic analysis of population health data Lancet 2006 367 1747 1757 10.1016/S0140-6736(06)68770-9 16731270

[B4] Roger VL Go AS Lloyd-Jones DM Adams RJ Berry JD Brown TM Heart disease and stroke statistics–2011 update: a report from the American Heart Association Circulation 2011 123 e18 e209 10.1161/CIR.0b013e3182009701 21160056 PMC4418670

[B5] Lange C Ziese T Daten und Fakten: Ergebnisse der Studie "Gesundheit in Deutschland aktuell 2009" 2010 Berlin: Robert Koch Institut

[B6] Löwel H Koronare Herzkrankheit und akuter Myokardinfarkt 2006 Berlin: Robert-Koch-Institut

[B7] Haggerty JL Reid RJ Freeman GK Starfield BH Adair CE McKendry R Continuity of care: a multidisciplinary review BMJ 2003 327 1219 1221 10.1136/bmj.327.7425.1219 14630762 PMC274066

[B8] Rosemann T Wensing M Rueter G Szecsenyi J Referrals from general practice to consultants in Germany: if the GP is the initiator, patients' experiences are more positive BMC Health Serv Res 2006 6 5 10.1186/1472-6963-6-5 16423299 PMC1386652

[B9] Wagner EH The role of patient care teams in chronic disease management BMJ 2000 320 569 572 10.1136/bmj.320.7234.569 10688568 PMC1117605

[B10] Lugtenberg M Burgers JS Westert GP Effects of evidence-based clinical practice guidelines on quality of care: a systematic review Qual Saf Health Care 2009 18 385 392 10.1136/qshc.2008.028043 19812102

[B11] Ho PM Magid DJ Shetterly SM Olson KL Maddox TM Peterson PN Medication nonadherence is associated with a broad range of adverse outcomes in patients with coronary artery disease Am Heart J 2008 155 772 779 10.1016/j.ahj.2007.12.011 18371492

[B12] Grimshaw JM Russell IT Effect of clinical guidelines on medical practice: a systematic review of rigorous evaluations Lancet 1993 342 1317 1322 10.1016/0140-6736(93)92244-N 7901634

[B13] Grimshaw J Eccles M Thomas R MacLennan G Ramsay C Fraser C Toward evidence-based quality improvement. Evidence (and its limitations) of the effectiveness of guideline dissemination and implementation strategies 1966–1998 J Gen Intern Med 2006 21 Suppl 2 S14 S20 16637955 10.1111/j.1525-1497.2006.00357.x PMC2557130

[B14] Perkins MB Jensen PS Jaccard J Gollwitzer P Oettingen G Pappadopulos E Applying theory-driven approaches to understanding and modifying clinicians' behavior: what do we know? Psychiatr Serv 2007 58 342 348 10.1176/appi.ps.58.3.342 17325107

[B15] Worrall G Chaulk P Freake D The effects of clinical practice guidelines on patient outcomes in primary care: a systematic review Canadian Medical Association Journal 1997 156 1705 1712 9220922 PMC1227585

[B16] Cabana MD Rand CS Powe NR Wu AW Wilson MH Abboud PA Why don't physicians follow clinical practice guidelines? A framework for improvement JAMA 1999 282 1458 1465 10.1001/jama.282.15.1458 10535437

[B17] Grol R Changing physicians' competence and performance: finding the balance between the individual and the organization J Contin Educ Health Prof 2002 22 244 251 10.1002/chp.1340220409 12613060

[B18] Lugtenberg M Zegers-van Schaick JM Westert GP Burgers JS Why don't physicians adhere to guideline recommendations in practice? An analysis of barriers among Dutch general practitioners Implement Sci 2009 4 54 10.1186/1748-5908-4-54 19674440 PMC2734568

[B19] Harrison MB Legare F Graham ID Fervers B Adapting clinical practice guidelines to local context and assessing barriers to their use CMAJ 2010 182 E78 E84 10.1503/cmaj.081232 19969563 PMC2817341

[B20] Kinsman L Rotter T James E Snow P Willis J What is a clinical pathway? Development of a definition to inform the debate BMC Med 2010 8 31 10.1186/1741-7015-8-31 20507550 PMC2893088

[B21] Vanhaecht K Bollman M Bower K Gallagher C Gardini A Prevalence and use of clinical pathways in 23 countries - an international survey by the European Pathway Association J Integr Care Pathw 2006 10 28 34

[B22] Ovretveit J The future for care pathways J Integr Care Pathw 2010 14 76 78 10.1258/jicp.2010.010009

[B23] Wood DA Kotseva K Connolly S Jennings C Mead A Jones J Nurse-coordinated multidisciplinary, family-based cardiovascular disease prevention programme (EUROACTION) for patients with coronary heart disease and asymptomatic individuals at high risk of cardiovascular disease: a paired, cluster-randomised controlled trial Lancet 2008 371 1999 2012 10.1016/S0140-6736(08)60868-5 18555911

[B24] Lelgemann M Ollenschlager G [Evidence based guidelines and clinical pathways: complementation or contradiction?] Internist (Berl) 2006 47 690 2 7 10.1007/s00108-006-1652-5 16763795

[B25] Silagy C Weller D Lapsely H Middleton P Shelby-James T Fazekas B The effectivenes of local adaptation of nationally produced clinical practice guidelines Family Practice 2002 19 223 230 10.1093/fampra/19.3.223 11978710

[B26] Sawicki PT Bastian H German health care: a bit of Bismarck plus more science BMJ 2008 337 a1997 10.1136/bmj.a1997 18996937

[B27] de Stampa M Vedel I Mauriat C Bagaragaza E Routelous C Bergman H Diagnostic study, design and implementation of an integrated model of care in France: a bottom-up process with continuous leadership Int J Integr Care 2010 10 e034 20216954 PMC2834925

[B28] Bergert F Braun M Conrad D Ehrenthal K Feßler J Gross J Hausärztliche Leitlinie Stabile Angina pectoris und KHK 2006 Köln: Leitliniengruppe Hessen

[B29] Nationale Versorgungsleitlinie Chronische KHK 2007 Köln: Deutscher Ärzte-Verlag

[B30] Craig P Dieppe P Macintyre S Michie S Nazareth I Petticrew M Developing and evaluating complex interventions: the new Medical Research Council guidance BMJ 2008 337 a1655 10.1136/bmj.a1655 18824488 PMC2769032

[B31] Sandelowski M Barroso J Classifying the findings in qualitative studies Qual Health Res 2003 13 905 923 10.1177/1049732303253488 14502957

[B32] Bourgeault I Dingwall R De Vries R (Eds) Qualitative methods in health research 2010 London: SAGE Publications Ltd

[B33] Sandelowski M Sample size in qualitative research Res Nurs Health 1995 18 179 183 10.1002/nur.4770180211 7899572

[B34] Hani MA Keller H Vandenesch J Sonnichsen AC Griffiths F Donner-Banzhoff N Different from what the textbooks say: how GPs diagnose coronary heart disease Fam Pract 2007 24 622 627 10.1093/fampra/cmm053 17971349

[B35] Keller H Kramer L Krones T Müller-Engelmann M Baum E Donner-Banzhoff N Evaluation der Implementierung von Innovationen am Beispiel von arriba - eine Fokusgruppenstudie Zeitschrift für Allgemeinmedizin 2011 87 35 41

[B36] Ajzen I Kuhl J, Beckmann J From intentions to actions: A theory of planned behavior Action control: From cognition to behavior 1985 New York: Springer 11 39

[B37] Maxqda 2007 Berlin: VERBI: Software Consult Sozialforschung GmbH

[B38] Mayring P Qualitative Inhaltsanalyse. Grundlagen und Techniken 2010 Weinheim: Beltz

[B39] Hunter DJ Fairfield G Managed Care: Disease management BMJ 1997 315 50 53 10.1136/bmj.315.7099.50 9233330 PMC2127038

[B40] Greenhalgh T Robert G Macfarlane F Bate P Kyriakidou O Diffusion of innovations in service organizations: systematic review and recommendations Milbank Q 2004 82 581 629 10.1111/j.0887-378X.2004.00325.x 15595944 PMC2690184

[B41] Ray-Coquard I Philip T de Laroche G Froger X Suchaud J Voloch A A controlled "before-after" study: impact of a clinical guidelines programme and regional cancer network organization on medical practice Br J Cancer 2002 86 313 321 10.1038/sj.bjc.6600057 11875690 PMC2375218

[B42] Formoso G Liberati A Magrini N Practice guidelines: useful and "participative" method? Survey of Italian physicians by professional setting Arch Intern Med 2001 161 2037 2042 10.1001/archinte.161.16.2037 11525707

[B43] Jones A A modernized mental health service: the role of care pathways J Nurs Manag 1999 7 331 338 10.1046/j.1365-2834.1999.00142.x 10827628

[B44] De Allegri M Schwarzbach M Loerbroks A Ronellenfitsch U Which factors are important for the successful development and implementation of clinical pathways? A qualitative study BMJ Qual Saf 2011 20 203 208 10.1136/bmjqs.2010.042465 21209137

[B45] Summerskill WS Pope C 'I saw the panic rise in her eyes, and evidence-based medicine went out of the door'. An exploratory qualitative study of the barriers to secondary prevention in the management of coronary heart disease Fam Pract 2002 19 605 610 10.1093/fampra/19.6.605 12429662

[B46] Murray E Treweek S Pope C MacFarlane A Ballini L Dowrick C Normalisation process theory: a framework for developing, evaluating and implementing complex interventions BMC Med 2010 8 63 10.1186/1741-7015-8-63 20961442 PMC2978112

[B47] Lemmens KM Nieboer AP Rutten-Van Molken MP van Schayck CP Spreeuwenberg C Asin JD Bottom-up implementation of disease-management programmes: results of a multisite comparison Qual Saf Health Care 2011 20 76 86 10.1136/bmjqs.2010.041236 21228079

[B48] Braithwaite J Runciman WB Merry AF Towards safer, better healthcare: harnessing the natural properties of complex sociotechnical systems Qual Saf Health Care 2009 18 37 41 10.1136/qshc.2007.023317 19204130 PMC2629006

[B49] Grimshaw JM Thomas RE MacLennan G Fraser C Ramsay CR Vale L Effectiveness and efficiency of guideline dissemination and implementation strategies Health Technol Assess 2004 8 1 72 10.3310/hta8060 14960256

[B50] Grol R Grimshaw J From best evidence to best practice: effective implementation of change in patients' care Lancet 2003 362 1225 1230 10.1016/S0140-6736(03)14546-1 14568747

[B51] Bronfenbrenner U The Ecology of Human Development: Experiments by Nature and Design 1979 Cambridge, MA: Harvard University Press

[B52] Sorensen G Emmons K Hunt MK Barbeau E Goldman R Peterson K Model for incorporating social context in health behavior interventions: applications for cancer prevention for working-class, multiethnic populations Prev Med 2003 37 188 197 10.1016/S0091-7435(03)00111-7 12914824

[B53] Vandenbroucke JP Observational research, randomised trials, and two views of medical science PLoS Med 2008 5 e67 10.1371/journal.pmed.0050067 18336067 PMC2265762

[B54] Campbell NC Murray E Darbyshire J Emery J Farmer A Griffiths F Designing and evaluating complex interventions to improve health care BMJ 2007 334 455 459 10.1136/bmj.39108.379965.BE 17332585 PMC1808182

[B55] Lewin S Glenton C Oxman AD Use of qualitative methods alongside randomised controlled trials of complex healthcare interventions: methodological study BMJ 2009 339 b3496 10.1136/bmj.b3496 19744976 PMC2741564

[B56] Paterson C Baarts C Launso L Verhoef MJ Evaluating complex health interventions: a critical analysis of the 'outcomes' concept BMC Complement Altern Med 2009 9 18 10.1186/1472-6882-9-18 19538715 PMC2712450

[B57] Bandura A Self efficacy: the exercise of control 1997 New York: Freeman

[B58] Carroll C Patterson M Wood S Booth A Rick J Balain S A conceptual framework for implementation fidelity Implement Sci 2007 2 40 10.1186/1748-5908-2-40 18053122 PMC2213686

[B59] Hasson H Systematic evaluation of implementation fidelity of complex interventions in health and social care Implement Sci 2010 5 67 10.1186/1748-5908-5-67 20815872 PMC2942793

[B60] Keith RE Hopp FP Subramanian U Wiitala W Lowery JC Fidelity of implementation: development and testing of a measure Implement Sci 2010 5 99 10.1186/1748-5908-5-99 21192817 PMC3161382

