# Supplementary material for: Qualitative evaluation of a local coronary heart disease treatment pathway: practical implications and theoretical framework
Source: BMC Fam Pract. 2012 May 14;13:36. doi: 10.1186/1471-2296-13-36 (PMC3489869; doi:10.1186/1471-2296-13-36)
Supplement: Additional file 1 — Pocket version of the CHD treatment pathway. [file 1471-2296-13-36-S1.pdf]

CHD Treatment Pathway — Medication

Improvement Prognosis

**Basic Medication**		
ASA	100 mg/d	Long-lasting
Simvastatin / Pravastatin	40 mg/d	Long-lasting
**+ status after acute coronary syndrome with/without intervention**		
Clopidogrel [1]	75 mg/d	(Dual inhibition!) 9 months,<br>after intervention with DES 1 year,<br>in case of ASA incompatibility long-lasting
Bisoprolol *or*<br>Metoprololsucc.	2.5 - 10 mg/d<br>23.75 - 190	After myocardial infarction long-lasting, also try in cases of COPD (only relative contraindication)
Ramipril [2]	2.5 - 10 mg/d	In cases of heart failure, possible in cases of hypertonia
**+ after elective stent**		
Clopidogrel [1]	75 mg/d	Dual inhibition for one month (BMS),<br>one year (DES);<br>If necessary duration of administration modified by interventionalist (e.g. anatomi-cal conditions)
**+ heart failure**		
Ramipril [2]	2.5 - 10 mg/d	Target dose 10 mg/d,<br>in cases of incompatibility Candesartan (target dose 32 mg/d)[3]
Bisoprolol [3]	2.5 -10 mg/d	Begin with 1.25 mg/d, gradually increase, target dose 10 mg/d
Spironolacton	25 mg/d	If symptomatic under ACE inhibitor + beta blocker treatment (50 mg tablet bisect)

1 : current status of authorization: day 1 – 28 Iscover® or Plavix®, then Clopidoprel generic

2 : Sartane only in case of ACE - inhibitors - incompatibility AND urgent ACE - inhibitor - indication (heart failure)

3 : *or* Nebivolol: in elderly + heart failure, 1.25mg to target dose 10 mg

4 : in case of symptomatic effective medication question indication quarterly, stop if necessary

Yearly influenza vaccination recommended.

Improvement Quality of life

**anti-anginal treatment[4]:**		
ISDN	10/20/40 mg<br>40/80/120 mg ret<br>per dose	Only in the mornings, if necessary also at noon-time
Molsidomin	1/2/4 mg<br>8 mg retard<br>per dose	If therapy is necessary in the evenings and/or at night
Bisoprolol ***or***	2.5 -10 mg/d	Simultaneous improvement of the prognosis
Metoprololsucc.	23.75 -190 mg	
**heart failure:**		
Furosemid	20-80 mg/d<br>(if so higher)	Short duration of drug effect
Torasemid	5-10 mg/d	Longer duration of drug effect
Xipamid	10-40 mg/d	(Thiazid) alone or in combination

Abbreviations:

PCI: percutaneous, coronary intervention
(= **PTCA**: percutaneous, transluminal, coronary angioplasty)

BMS : bare metal stent, uncoated stent

DES : drug eluting stent, (drug -)coated stent

UCG: ultrasound cardiograph, heart echo

LVEF: left ventricular ejection fraction, cardiac output

SE: side effects

BP: blood pressure

Regular care stable CHD

	GP	Cardiologist
every 6 months	BP control, physical examination, weight, medication check<br>Quality of life? / Behaviour?<br>Labour not obligatory, except K, crea under ACE inhibitor/AT-1 inhibitor/ potassium-sparing diuretics<br>Muscle pain under statins?	

Physical examination: Heart auscultation, lung, leg oedemas

Quality of life: Coping with everyday life, sexuality, joy of life, social contacts, fears

Depression:
1. Did you feel down, depressed or hopeless in the last month?
2. During the last month, did you have less interest or joy in things that you used to like?

Behaviour: Smoking, intensity/lack of motions, over-/malnurishment
Did you take your drugs as recommended?

Elective PCI (PTCA) +/- Intervention (mostly) without Rehab

	GP	Cardiologist
after intervention	Check medication,<br>enroll in a DMP<br>Quality of life? / Behaviour?<br>If necessary cardiac rehab groups<br>Incapacity for work: 3 – 5 days (medically sufficient)	
4 weeks		Exercise ECG (in-stent-stenosis?)
3 months	BP control, physical examination, weight, medication check / SE?<br>Quality of life? / Behaviour?<br>Labour: total chol, LDL; statins new: CK, GPT; under ACE inhibitor/AT-1 inhibitor/ potassium-sparing diuretics: K, crea	
6 months	Findings documentation to the cardiologist	Exercise ECG (if pathologic before PCI)<br>UCG (optional)
12 months +<br>every 6 months	BP control, physical examination, weight, medication check<br>Quality of life? / Behaviour?<br>Labour not obligatory, except K, crea under ACE inhibitor/AT-1 inhibitor/ potassium-sparing diuretics	In special cases control exercise ECG after 12 months,<br>Announcement by the cardiologist
3 years		Exercise ECG , UCG (optional)

After Acute Coronary Syndrome (ACS) / Bypass + (generally) Rehab

	GP	Cardiologist
after discharge	Medication check, muscle pain?<br>enroll in a DMP<br>Quality of life? / Behaviour?	
3 months		Medication check<br>Exercise ECG , UCG
6 months	BP control, physical examination, weight, medication check / SE?<br>Quality of life? / Behaviour?<br>Labour: total chol / LDL; under ACE inhibitor/AT-1 inhibitor/ potassium-sparing diuretics: K, crea	
12 months		Exercise ECG , UCG (optional)
1 year +<br>every 6 months	BP control, physical examination, weight, medication check<br>Quality of life? / Behaviour?<br>Labour not obligatory	
3 years		Exercise ECG , UCG (optional)

Additional contact in case of disorders, unclear situation etc. every time!

CHD and heart failure

	GP	Cardiologist
**LVEF ≥ 50**	GP care like stable CHD	
**LVEF 35 - 50**<br>every 6 – 12 weeks	BP control, physical examination, weight, medication: intake / SE?<br>check weight protocol<br>Quality of life? / Behaviour?<br>Labour not obligatory; under ACE inhibitor/AT-1 inhibitor/ potassium-sparing diuretics: K, crea	
every 12 months	**Info Cardiologist:** patient, weight, labour, BP / pulse, medication,	ECG, possibly 24h – ECG, UCG
**LVEF ≤ 35**<br>every 6 weeks	BP control, physical examination, weight, medication: intake / SE?<br>check weight protocol<br>Quality of life? / Behaviour?<br>Labour: K, crea, GPT (ALT)	
every 6 weeks	**Info Cardiologist:** patient, weight, labour, BP / pulse, medication, specialty	ECG, possibly 24h – ECG, UCG
